# Contextualizing Help-Seeking Attitudes and Help-Seeking Intention: The Role of Superwoman Schema among Black College Women

**DOI:** 10.1007/s40615-024-02075-0

**Published:** 2024-07-01

**Authors:** Tamara Nelson, Samrawit B. Gebretensay, Andrea M. Sellers, Oswaldo Moreno

**Affiliations:** 1 Department of Psychology, Rutgers University Camden, 311 N. Fifth Street, Camden, NJ 08102 USA; 2 Department of Psychology, Virginia Commonwealth University, 806 W. Franklin St., Richmond, VA 23284 USA

**Keywords:** Help-seeking attitudes, Help-seeking intention, Superwoman schema, Black women

## Abstract

Black women are less likely to seek psychological help and underutilize mental health services. Although help-seeking attitudes and intentions are associated in the general population, less is known about this relationship among Black women in college. In this cross-sectional study, we investigated the relationship between help-seeking attitudes and intention among 167 self-identified Black women in college. We also investigated if dimensions of the Superwoman Schema (i.e., an obligation to display strength, resistance to being vulnerable, an obligation to suppress emotions, an intense motivation to succeed despite limited resources, and an obligation to help others) moderated this relationship. Findings indicated a significant positive relationship between help-seeking attitudes and help-seeking intention. Regarding moderation, an obligation to suppress emotions, resistance to vulnerability, and an obligation to help others interacted with help-seeking attitudes in predicting help-seeking intention. Notably, low adherence to an obligation to suppress emotions, resistance to vulnerability, and an obligation to help others were associated with high levels of help-seeking intention. However, more favorable help-seeking attitudes improved help-seeking intention for participants high in adherence to these dimensions. Our findings suggest that understanding the relevance of the Superwoman Schema among Black women is critical for increasing help-seeking behavior.

In a nationally representative sample of college students (i.e., National Healthy Minds Study), 50.4% of Black college students reported clinically significant symptoms of one or more mental health conditions (e.g., depression, anxiety, nonsuicidal self-injury) during 2020–2021; yet only 30% reported receiving mental health treatment [1]. Generally, Black college students underutilize mental health services and are reluctant to seek help partly due to negative experiences associated with the help-seeking process [2, 3]. Additionally, cultural mistrust, stigma, discrimination, spiritual practices, beliefs, and reliance upon informal sources of support explain less use of mental health treatment [3–9]. Moreover, attitudes about help-seeking for mental health tend to be negative in this population [10–12]. As help-seeking attitudes predict help-seeking intention in the general population [13], one study found that help-seeking attitudes did not predict help-seeking intention among Black college students [14]. Thus, it is crucial to examine these relationships among Black college women who experience unique stressors at the intersection of racism and sexism that predict psychological distress [15–18].

To counter stressors, some Black college women may cope by adhering to gendered racialized roles, such as Superwoman Schema (SWS), which may help and hinder the help-seeking process [19–22]. SWS is a culturally salient phenomenon operationalized as an obligation to display strength, resistance to being vulnerable, an obligation to suppress emotions, an intense motivation to succeed despite limited resources, and an obligation to help others [20, 22, 23]. SWS is associated with both positive (i.e., resilience, pride) and negative outcomes (i.e., anxiety, depression, stress, and decreased help-seeking [21, 22, 24–27]. Thus, investigating SWS is critical to understanding mental health treatment-seeking behaviors. In this study, we examined the relationship between help-seeking attitudes and help-seeking intention. We also examined the potential moderating role of the Superwoman Schema.

## Planned Behavior Theory

A theoretical model of behavioral change may help to understand the complexity of mental health help-seeking behavior [28] in this population. According to Planned Behavior Theory [29] help-seeking intention (i.e., the intention to seek help from a mental health professional when having a mental health concern) [30] is the most robust determinant of help-seeking behavior [29]. Help-seeking intention depends on an individual’s attitudes toward help-seeking, the opinions of others in the individual's social environment, and the perceived barriers to treatment [28, 29, 31]. While findings from previous research in the general population have indicated that recognition of the need for help is associated with help-seeking intention [32] another study found that attitudes were the strongest predictor of intention to seek mental health services [33]. Taken together, investigating potential factors that shape help-seeking attitudes may be critical for a deeper understanding of help-seeking intention [3]. A deeper understanding of these processes may result in targeted areas for intervention and decrease the gap in service use in this population [3].

## Help-Seeking Attitudes and Intention among Black College Women

There are significant disparities in mental healthcare utilization: Black Americans tend to seek and receive less mental healthcare treatment than their White counterparts [34–38]. Several factors (i.e., affordability, cultural mistrust, discrimination, and stigma) account for these differences [8, 9, 39]. For example, in previous research, Black college students have reported seeking help from myriad sources including professional clinicians, roommates, friends, significant others, family members, religious counselors, support groups, or other non-clinical sources [40, 41]. Nonetheless, Black college students typically use informal support networks [40, 41]. The reliance upon informal sources of support may be due in part to the lack of culturally sensitive providers and past negative experiences with mental healthcare treatment [3, 42]. Indeed, in a study of Black Americans’ experiences of mental health treatment and providers, some reported negative experiences with providers noting that they were unhelpful, insensitive, and in some cases harmful [42]. However, in another study among Black college women, past mental health treatment buffered the negative association between psychological distress and help-seeking attitudes, suggesting a potential protective effect of past mental health treatment [43].

The help-seeking process for psychological distress is informed by both past experiences with mental treatment and help-seeking attitudes [2]. Help-seeking attitudes are a significant predictor of mental healthcare utilization [13, 35, 37, 42] and depend on the complex interplay of perceiving the need for psychological support, navigating stigma associated with seeking help, being open to sharing personal struggles, and having confidence in the effectiveness of mental health professionals [11, 44]. Notably, findings from several studies have indicated that Black college students tend to have unfavorable attitudes toward mental healthcare utilization [10, 11, 45–47]. In other studies, help-seeking attitudes have been positive [48, 49] and mixed [4, 50]. Yet, despite research examining help-seeking processes among Black college students, very few studies have examined help-seeking attitudes and help-seeking intention simultaneously, let alone how cultural factors, such as Superwoman Schema might impact this relationship.

Utilizing the Theory of Planned Behavior as a framework, researchers explored Black Americans' help-seeking intentions, identifying key factors deterring them from seeking help [3]. Barriers included stigma, societal pressure against seeking help, and perceived challenges like mistrust and discrimination in therapy [3]. Further, it was noted that additional research exploring the interaction between culturally salient phenomena and help-seeking intention was needed [3]. In another study, researchers examined help-seeking intention in a nationally representative sample of Black college students who were diagnosed with depression. Help-seeking intention was measured by the question, “If you were experiencing serious emotional distress, whom would you talk to about this” [41]. Participants responded to nine types of help-seeking sources including mental health providers, family, and friends, as well as no one, which were categorized into a binary variable (i.e., yes/no) that measured help-seeking intention [41]. Findings revealed that 89.4% of the sample reported help-seeking intention; Black college women were more likely to report help-seeking intention than their male counterparts; and there was a positive association between flourishing (i.e., self-perception of psychological well-being and social connectedness) [51] and help-seeking intention [41].

Research on mental health help-seeking processes among Black college women is increasing, which has the potential to address low mental health treatment utilization [1, 3–5]. However, a critical gap remains in understanding the relationship between attitudes toward mental health help-seeking and the actual intention to seek help among Black women. This gap hinders effective interventions and leaves Black college women at heightened risk of facing untreated mental health concerns [3, 43]. Thus, unpacking this complex interplay between attitudes, intentions, and cultural influences like the Superwoman Schema is crucial for developing culturally competent interventions that bridge the gap between positive attitudes and actual help-seeking behavior among Black college women.

## The Moderating Role of Superwoman Schema

Superwoman Schema (i.e., an obligation to display strength, resistance to being vulnerable, an obligation to suppress emotions, an intense motivation to succeed despite limited resources, and an obligation to help others) has been generally described as how Black women are expected to perform and embody womanhood [20–25, 52–55]. Some dimensions of SWS have been associated with anxiety, depression, and perceived stress [22, 25, 26, 56]. Others have been associated with resilience (i.e., assets associated with resilient outcomes) and self/ethnic pride (i.e., self-confidence as a Black woman) [20, 24, 25]. Additionally, SWS may be culturally relevant for understanding and intervening in help-seeking attitudes and intentions. Several findings from qualitative studies have suggested that adherence to this phenomenon may prevent Black women from asking for help, showing vulnerability, and emotional expression [4, 19–21, 54]. Apart from Watson and Hunter’s (2015) study, where the authors found an inverse association between the Strong Black Woman Schema (i.e., a construct akin to SWS, but measured differently) and psychological openness and help-seeking propensity in a sample of 95 Black women [19], we are unaware of published studies exploring this phenomenon. Further, more research is needed to understand which dimensions of SWS might predict help-seeking intention, as little research has been conducted to directly evaluate these relationships [4].

## The Current Study

Superwoman Schema has been less explored when investigating mental health services utilization [20, 22]. While help-seeking attitudes are associated with help-seeking intention in the general population, less is known about this relationship in Black college women. To address this gap, we examined the relationship between help-seeking attitudes and intention in a sample of Black college women. We also examined if dimensions of the Superwoman Schema moderated this relationship. We predicted that help-seeking attitudes would be positively associated with help-seeking intention and that Superwoman Schema would moderate this relationship.

## Methods

### Participants

The sample for the current study included 167 self-identified Black college women. Participants were ages 18 to 54, and the mean age of the sample was 22.20 (*SD* = 5.83). Over half of the participants reported being first-generation college students (55%), and 17% of the sample reported being born outside of the United States. The distribution of participants by class year was: 27% first-year, 17% sophomores, 27% juniors, 18% seniors, and 11% graduate or doctoral students. Most of the sample reported being heterosexual (72%), and 28% identified as lesbian, gay, bisexual, or other. Almost 43% of the sample reported receiving mental health treatment (i.e., counseling or therapy) for a mental health condition.

### Measures

#### Help-Seeking Attitudes

Help-seeking attitudes were assessed using the Mental Help-Seeking Attitudes Scale (MHSAS; [57]). The MHSAS consists of 9 items that comprise a unidimensional assessment of an individual’s help-seeking from a mental health professional if experiencing a mental health concern. Participants responded to items on a seven-point Likert scale ranging from 1 to 7. Mean scores were calculated by adding scores for all nine items and dividing by nine. Higher MHSAS scores indicate more positive attitudes toward mental health help-seeking. The MHSAS has excellent psychometric properties, reliability, and validity [56]. The Cronbach’s alpha for the current study was 0.89.

#### Help-Seeking Intention

Help-seeking intention was assessed using the Mental Help-Seeking Intention Scale (MHSIS; [30]. The MHSIS contains 3 items that provide a unidimensional assessment of an individual’s help-seeking intention from a mental health professional if experiencing a mental health concern. Participants responded to items on a seven-point Likert scale ranging from 1 to 7. Mean scores were calculated by adding scores for all three items and dividing by three. Higher scores indicate an increased intention to seek help. The MHSIS has excellent psychometric properties, reliability, and validity [30]. The Cronbach’s alpha for the current study was 0.92.

#### Superwoman Schema

Superwoman Schema (SWS) was assessed using the Giscombé Superwoman Schema Questionnaire (G-SWS-Q; [22]). Based on Woods-Giscombé’s (2010) qualitative research with Black women [20], the G-SWS-Q consists of 35 items that measure five dimensions of the SWS, including an obligation to present an image of strength (6 items), an obligation to suppress emotions (7 items), resistance to being vulnerable (7 items), an intense motivation to succeed, despite limited resources (6 items), and an obligation to help others (9 items). Participants responded to items on a 4-point scale ranging from 0 = not true for me to 3 = true for me all the time. Higher SWS scores indicate greater identification with SWS dimensions. The G-SWS-Q has demonstrated strong psychometric properties, including a clear factor structure, high internal consistency, and construct validity [22]. The Cronbach’s alpha for the current study was 0.74 for an obligation to present an image of strength, 0.83 for an obligation to suppress emotions, 0.84 for resistance to being vulnerable, 0.74 for an intense motivation to succeed, despite limited resources, and 0.84 for an obligation to help others.

#### Demographics

Participants provided information regarding age, sexual orientation, generation status, nationality, class year, and experience with mental health treatment.

### Procedure

The research described in this study received approval from the institutional review board (IRB) of the first author’s institution. Participants were recruited through the Psychology Department’s human research pool at a public university minority-serving institution. Recruitment also included contacting multicultural centers, African American/Black student organizations and affinity groups, and African American Studies programs via email notification. Participants interested in the study were asked to contact the study team using the provided QR code or email address with their institutional email address to receive the link and password to the web-based survey in Qualtrics. After reviewing the informed consent, participants affirmed consent to continue to the next page containing the survey. The survey included the measures and a demographic questionnaire. Participants received a $10 Amazon electronic gift card upon study completion as an incentive. We used a separate incentive database to avoid linking participants’ provided emails to receive the incentive with their submitted survey.

### Analytic Approach

We examined normality, skewness, and kurtosis for all predictors and outcomes in this study. All variables were deemed fit for analysis. Next, we conducted several one-way analyses of variance (ANOVA) models to determine if there were statistically significant differences in help-seeking intention by all demographic characteristics. We controlled for any statistically significant predictor of help-seeking intention in subsequent analyses. We ran bivariate Pearson correlations to investigate the relationship between age, measured as a continuous variable, predictors, outcomes, and moderators. Finally, we conducted a series of 5 separate moderation analyses, controlling for past mental health treatment with help-seeking intention as the dependent variable, help-seeking attitudes as the independent variable, and dimensions of Superwoman Schema as the moderators. For all moderation analyses, we used 5,000 bootstrap estimates. Interactions were probed if *p* < 0.05 with conditioning values of the predictor at one standard deviation above and below the mean. All statistical analyses were conducted in SPSS Version 28.0; moderation analyses, including syntax and data for probing and visualizing interactions, were generated in Hayes’ PROCESS Macro Version 4.2 [58, 59].

## Results

### Preliminary Analyses

Sample means standard deviations, correlations, and reliability for all study variables are provided in Table 1. Help-seeking intention differed only by past mental health treatment. Notably, there were significantly higher levels of help-seeking intention among those who reported past mental health treatment (*M* = 5.67, *SD* = 1.61) compared to those who did not report past mental health treatment (*M* = 4.35, *SD* = 1.63). Bivariate Pearson correlations revealed significant associations between help-seeking attitudes and help-seeking intention (*r* = 0.36, *p* < 0.001). None of the Superwoman Schema dimensions were associated with help-seeking attitudes. However, there were significant inverse associations for emotion suppression (*r* = -0.26, *p* < 0.001) and resistance to vulnerability (*r* = -0.27, *p* < 0.001) on help-seeking intention. There were no significant associations between age, dimensions of the Superwoman Schema, help-seeking attitudes, or intention.

**Table 1:** Summary of means, standard deviations, correlations, and reliability for study variables

Variable	*M*(*SD*)	1	2	3	4	5	6	7	8	*α*
1. Age	22.20 (5.83)									
2. Obligation to Present Strength	13.68 (3.35)	.06								.74
3. Emotion Suppression	14.65 (4.47)	−.13	.42***							.83
4. Resistance to Vulnerability	15.20 (4.34)	.01	.45***	.66***						.84
5. Intense Motivation to Succeed	14.10 (3.13)	.09	.55***	.43***	.49***					.74
6. Obligation to Help Others	17.15 (5.89)	.09	.50***	.54***	.56***	.54***				.84
7. Help-Seeking Attitudes	5.37 (1.27)	.08	.05	−.10	−.02	.11	−.04			.89
8. Help-Seeking Intention	4.85 (1.75)	.21	−.02	−.26***	−.27***	−.03	−.08	.36***		.92

^*^p < .05, **p < .01, ***p < .001

### Moderation Analyses

**Fig. 1:**
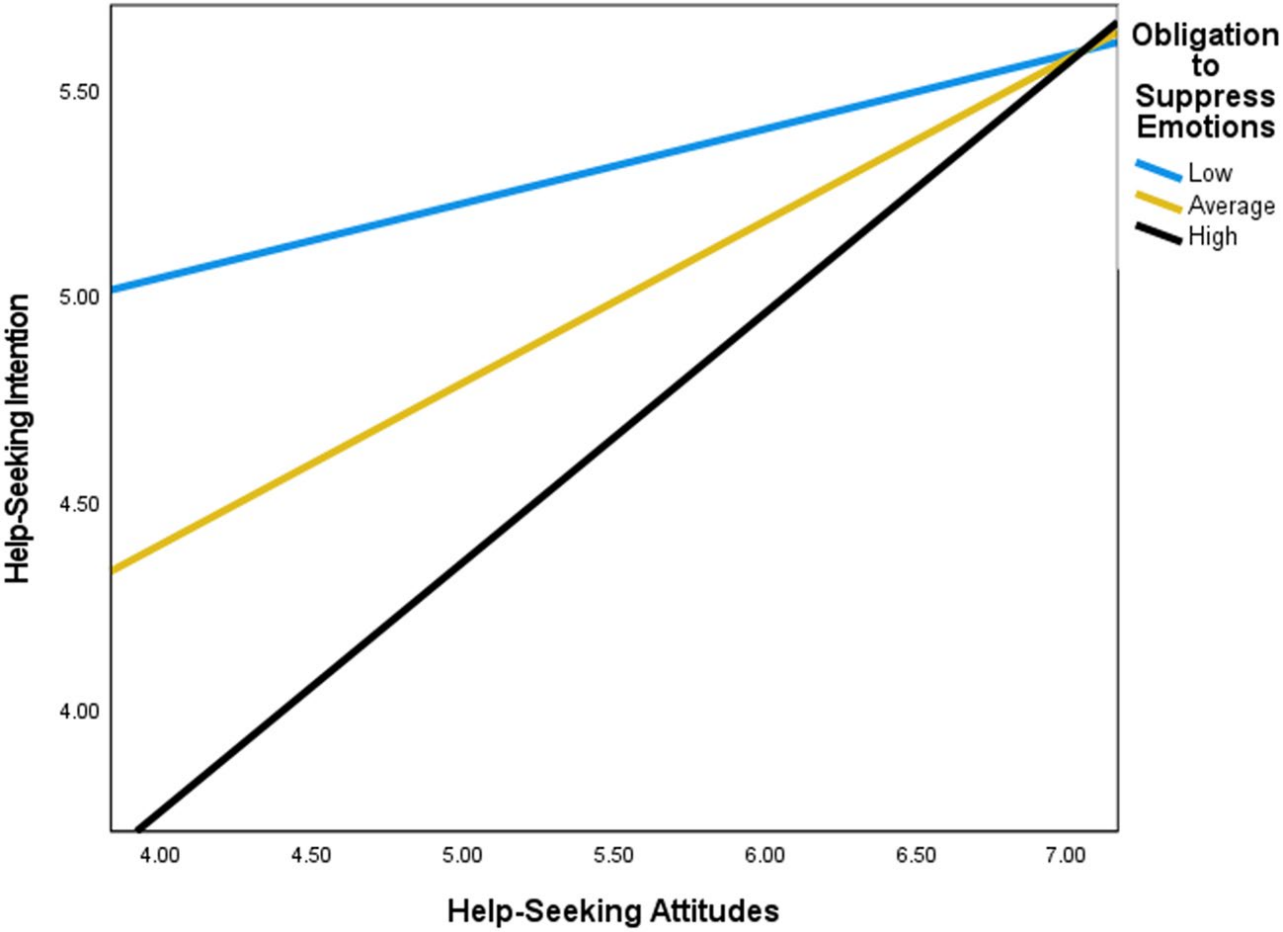
Moderation of help-seeking attitudes on help-seeking intention by obligation to suppress emotions

**Fig. 2:**
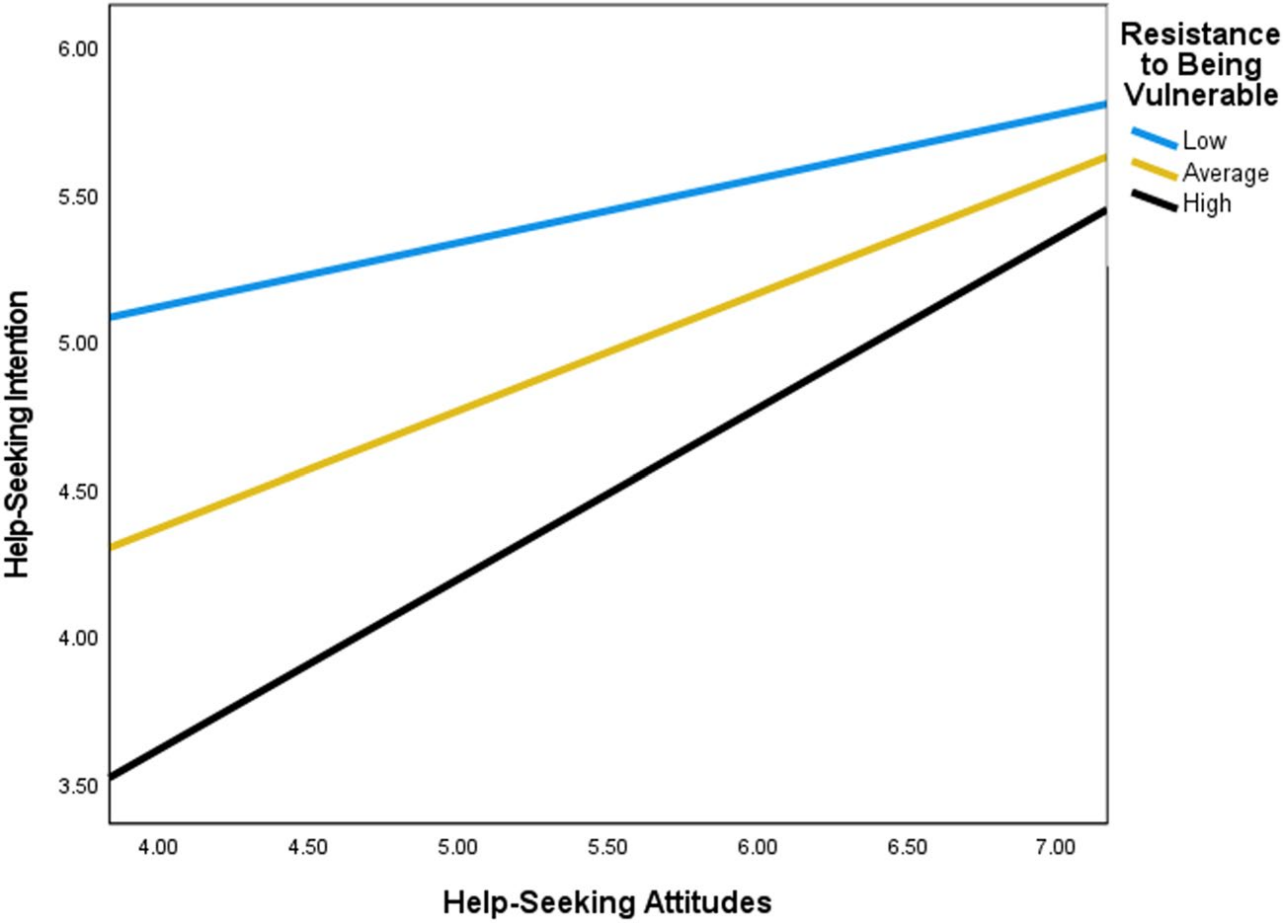
Moderation of help-seeking attitudes on help-seeking intention by resistance to vulnerability

**Fig. 3:**
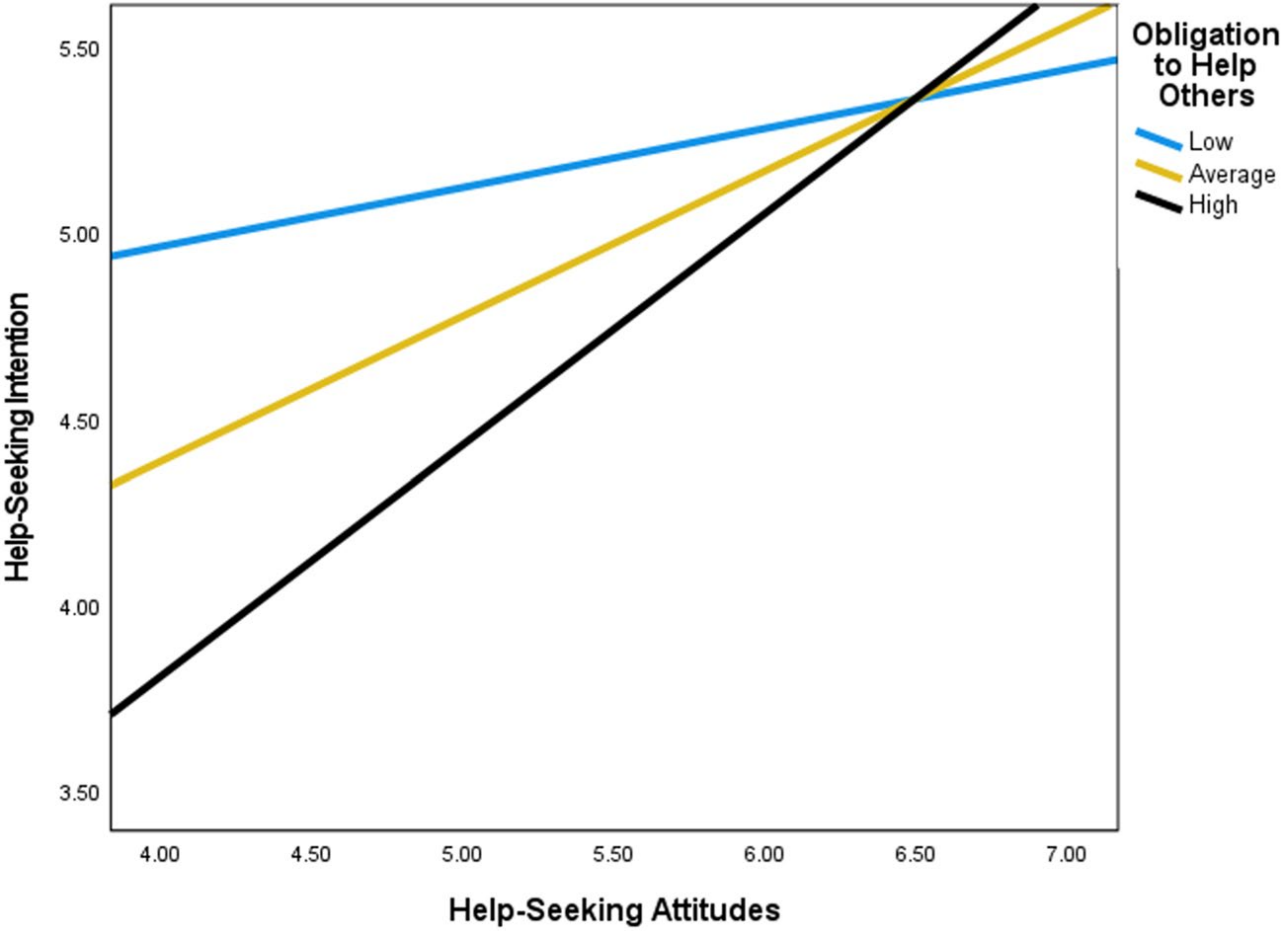
Moderation of help-seeking attitudes on help-seeking intention by an obligation to help others

Moderation analyses indicated significant interactions between three dimensions of SWS (i.e., an obligation to suppress emotions, resistance to vulnerability, and an obligation to help others) and help-seeking attitudes in predicting help-seeking intention (see Table 2 and Figs. 1, 2, and 3). Overall, findings revealed that high adherence to the SWS dimensions, an obligation to suppress emotion, resistance to vulnerability, and an obligation to help others, suppressed help-seeking intention at low levels of help-seeking attitudes. Accordingly, the steepest inclines in the slopes for help-seeking intention when help-seeking attitudes were more favorable, were observed for Black women with high levels of an obligation to suppress emotion, resistance to vulnerability, and an obligation to help others. Finally, high levels of help-seeking attitudes reduced the negative impact of an obligation to suppress emotions, resistance to vulnerability, and an obligation to suppress emotions on help-seeking intention.

**Table 2:** Help-seeking attitudes and help-seeking intention moderated by dimensions of superwoman schema

	Help-seeking intention		
	*B* (*SE*)	*t*	*p*
Help-Seeking Attitudes	.30 (.40)	.75	.451
Obligation to Present Strength	−.10 (.16)	−.65	.516
Help-Seeking Attitudes * Obligation to Present Strength	.01 (.03)	.29	.772
Help-Seeking Attitudes	−.29 (.33)	−.87	.385
Emotion Suppression	−.33 (.12)	−2.74	.007
Help-Seeking Attitudes * Emotion Suppression	.05 (.02)	2.14	.034
Help-Seeking Attitudes	−.22 (.32)	−.70	.484
Resistance to Vulnerability	−.33 (.11)	−3.06	.003
Help-Seeking Attitudes * Resistance to Vulnerability	.04 (.02)	2.04	.042
Help-Seeking Attitudes	−.31 (.43)	−.72	.475
Intense Motivation to Succeed	−.35 (.17)	−2.04	.043
Help-Seeking Attitudes * Intense Motivation to Succeed	.05 (.03)	1.73	.085
Help-Seeking Attitudes	−.30 (.31)	−.97	.330
Obligation to Help Others	−.26 (.09)	−2.80	.006
Help-Seeking Attitudes * Obligation to Help Others	.04 (.02)	2.38	.018

^*^Adjusted for past mental health treatment

## Discussion

This study examined the relationship between help-seeking attitudes and intention in a sample of Black college women; this study is also the first to examine the moderating role of SWS (i.e., an obligation to display strength, resistance to being vulnerable, an obligation to suppress emotions, an intense motivation to succeed despite limited resources, and an obligation to help others) on the relationship between help-seeking attitudes and intention. Consistent with the literature, findings revealed significant associations between help-seeking attitudes and help-seeking intention. Of the five dimensions of SWS, only three (i.e., an obligation to suppress emotions, resistance to vulnerability, and an obligation to help others) moderated the relationship between help-seeking attitudes and help-seeking intention. An obligation to manifest strength and an intense motivation to succeed, despite limited resources did not moderate these relationships.

The moderating role of Superwoman Schema dimensions in the relationship between help-seeking attitudes and intentions increases our understanding of the complex nature and salience of this construct in the lives of Black women. Notably, high levels of an obligation to suppress emotions, resistance to vulnerability, and an obligation to help others suppressed help-seeking intention at low levels of help-seeking attitudes in this sample. Thus, our findings demonstrate the importance and influence of SWS on help-seeking attitudes and intention among Black college women. Notably, help-seeking intention was high among participants, with a low endorsement of an obligation to suppress emotions, resistance to vulnerability, and an obligation to help others, despite limited resources. For participants with average and high levels of SWS dimensions, as help-seeking attitudes improved, help-seeking intention also significantly increased, as evidenced by the steep slopes. Thus, our findings add to existing literature documenting negative outcomes associated with adherence to Superwoman Schema in help-seeking. We have identified three key areas to inquire about and address to enhance help-seeking and treatment utilization in this population. Consequently, this expresses an immediate need to increase efforts that address help-seeking attitudes contextually vis-a-vis Superwoman Schema.

For example, the unspoken obligation to suppress emotions, resistance to vulnerability, and an obligation to help others, despite limited resources may create challenges in recognizing psychological distress, perceiving the need to address this distress, and willingness to seek mental health treatment among Black college women [4]. This is important as recognition that one needs help is associated with help-seeking intention [32]. In other words, Superwoman Schema may play a unique role in shaping help-seeking attitudes. Further, the negative impact on help-seeking intention may be more pronounced among college Black women who adhere strongly to these three dimensions. Conversely, when adherence is high, improving help-seeking attitudes may benefit this population, resulting in increased help-seeking intention. Taken together, our findings allude to the complexity of Superwoman Schema as a unique phenomenon that is salient and important for addressing mental health and treatment seeking in the lives of Black women.

### Clinical, Research, and Policy Implications

Findings also suggest the importance of dimensions of the Superwoman Schema in conceptualizing help-seeking attitudes and intention in practice, research, and policy. First, clinicians mindful of how adherence to Superwoman Schema dimensions may hinder help-seeking among Black college women may be uniquely positioned to enhance treatment engagement. Contextualizing Superwoman Schema, as a gendered racialized role in response to oppression [21–23], may assist clinicians in tailoring outreach messages to increase help-seeking in this population. In addition, understanding the potential salience of Superwoman Schema in Black women's lives might aid clinicians in decreasing internalized stigma toward help-seeking, normalizing vulnerability, and facilitating self-care [19, 20, 24, 54]. Finally, accessible, and affordable mental health providers that are culturally humble and knowledgeable about SWS have the potential to address concerns in this population more adequately.

Second, to address the increased prevalence of mental health disorders among college students, in a recent study, researchers formulated a comprehensive argument for expanding prevention programs to incorporate help-seeking intentions and behaviors as explicit outcomes [60]. The authors proposed that examining these outcomes can enhance effectiveness and result in a prolonged impact of programs among college students [60]. We agree and contend that researchers investigating the help-seeking process among Black college women include measures of SWS [22]. As SWS is culturally salient and associated with help-seeking intentions and interacts with help-seeking attitudes, we propose that targeting dimensions of SWS can potentially develop culturally tailored messages in prevention and intervention efforts for this population. Cultural adaptations of help-seeking interventions and empirically supported treatments that infuse SWS should also be investigated to provide evidence of the potential effectiveness of these interventions in improving the mental health of college Black women. Additionally, researchers conducting longitudinal studies investigating these constructs, as well as mechanisms that explain the relationships between these constructs, have the potential to enhance understanding of the help-seeking process for college Black women.

Finally, universities need to recognize the unique barriers that Black women in college face regarding mental health. Universities must prioritize the hiring of mental health professionals who are culturally humble to increase access and provide better mental health services for Black women in college. Moreover, creating a more supportive and inclusive environment and a culture of support and openness around mental health so that Black women in college will feel comfortable seeking help has the potential to increase treatment utilization and engagement. Universities should develop and implement campus policies addressing discrimination, mental health stigma, and barriers to seeking mental health treatment. Additionally, counseling centers should provide training and resources to faculty and staff to increase mental health awareness in order to effectively recognize and address these issues. This comprehensive approach could equip college Black women to prioritize their well-being and improve the mental health help-seeking process.

### Limitations and Strengths

There are several limitations to this study worth noting. First, this study includes a convenience sample of Black college women in the Northeast region of the United States. Consequently, generalizability to Black women outside of this population is limited. Second, participants provided self-reported data, which may result in overreporting or underreporting variables of interest in this study (e.g., help-seeking attitudes, help-seeking intention, dimensions of Superwoman Schema). Moreover, research findings from self-reported data may not accurately reflect reality. This can lead to unreliable comparisons, particularly when studying groups like Black women where reporting tendencies might differ based on geographic region, socioeconomic status, or other demographics. Third, this study is cross-sectional and descriptive. Thus, we cannot address causality or make any inferences about the directionality or longitudinal nature of these associations. Finally, we do not have specific information about help-seeking behaviors above and beyond help-seeking attitudes and intention. Thus, these associations are speculative about the predictive nature of Black college women’s willingness to seek psychological treatment for mental health conditions.

Despite these limitations, this study has several strengths. First, we focused on a sample of diverse Black college women attending a large public institution. More than half the sample identified as first-generation college students; nearly 30% identified as a sexually minoritized person; and 17% were born outside of the United States. This is important as previous studies on help-seeking attitudes include aggregate college student data, which may not adequately capture the unique experiences of Black women. Second, we focused on college Black women’s psychological help-seeking attitudes and help-seeking intention. Given the increased need to understand how factors such as SWS may influence help-seeking in this population, we deem this important. Moreover, findings from this study extend previous research [14] by including a culturally salient construct (i.e., SWS) that may elucidate the nature of subjective norms experienced by Black college women. They might be relevant for understanding perceived behavioral control, thus elaborating on the theory of planned behavior. Future research studies would include measures of perceived behavioral control and ask about the familial, societal, and communal pressure to adhere to Superwoman Schema dimensions (e.g., subjective norms).

### Conclusion

The mental health of Black college students is critically important, given the increasing prevalence of mental health conditions and low rates of mental health treatment utilization. Consequently, this population must address structural inequities that underscore mental health care disparities coupled with increased awareness, destigmatization, and prioritization of enhancing mental health treatment. Additionally, future research that includes contextual factors associated with help-seeking in this population can enhance public health messaging and preventive efforts that promote self-care and increased treatment utilization within this population.
